# The Involvement of Protease Nexin-1 (PN1) in the Pathogenesis of Intervertebral Disc (IVD) Degeneration

**DOI:** 10.1038/srep30563

**Published:** 2016-07-27

**Authors:** Xinghuo Wu, Wei Liu, Zhenfeng Duan, Yong Gao, Shuai Li, Kun Wang, Yu Song, Zengwu Shao, Shuhua Yang, Cao Yang

**Affiliations:** 1 Department of Orthopaedic Surgery, Union Hospital, Tongji Medical College, Huazhong University of Science and Technology, Wuhan 430022, China; 2 Department of Orthopaedic Surgery, Massachusetts General Hospital and Harvard Medical School, Boston, MA, USA.

## Abstract

Protease nexin-1 (PN-1) is a serine protease inhibitor belonging to the serpin superfamily. This study was undertaken to investigate the regulatory role of PN-1 in the pathogenesis of intervertebral disk (IVD) degeneration. Expression of PN-1 was detected in human IVD tissue of varying grades. Expression of both PN-1 mRNA and protein was significantly decreased in degenerated IVD, and the expression levels of PN-1 were correlated with the grade of disc degeneration. Moreover, a decrease in PN-1 expression in primary NP cells was confirmed. On induction by IL-1β, the expression of PN-1 in NP cells was decreased at day 7, 14, and 21, as shown by western blot analysis and immunofluorescence staining. PN-1 administration decreased IL-1β-induced MMPs and ADAMTS production and the loss of Agg and Col II in NP cell cultures through the ERK1/2/NF-kB signaling pathway. The changes in PN-1 expression are involved in the pathogenesis of IVD degeneration. Our findings indicate that PN-1 administration could antagonize IL-1β-induced MMPs and ADAMTS, potentially preventing degeneration of IVD tissue. This study also revealed new insights into the regulation of PN-1 expression via the ERK1/2/NF-kB signaling pathway and the role of PN-1 in the pathogenesis of IVD degeneration.

Low-back pain (LBP) is among the leading causes of the most costly musculoskeletal problems in adults worldwide. Intervertebral disc degeneration (IDD) is one of the most common disorders reported in orthopedic practice resulting in LBP; the magnitude of the issue is intensified by the increasing adult population, attaining an overall cost exceeding $100 billion per year in the United States^1^,^2^. Advances in the research on disc physiology and the etiology of IDD have been made, and a strong association between IDD and LBP has been shown^3^,^4^. The pathogenesis of IDD is very complicated and remains poorly understood. To date, possible etiological factors in the pathogenesis of IDD have been identified as aberrant, cell-mediated, age- and genetic-dependent molecular degeneration processes^5^.

An IVD consists mainly of a central nucleus pulposus (NP) and radially aligned annulus fibrosus (AF), both of which play a key role in spinal column articulation, force coordination, and cushioning against axial load^6^. During degeneration, the composition and structure of the IVD are altered, resulting in impaired biomechanical function^7^,^8^. Papers published in the medical literature^9^,^10^,^11^,^12^,^13^ have emphasized that the expression of matrix metalloproteinases (MMPs) and the disintegrin and metalloproteinase with thrombospondin motifs (ADAMTS) genes have essential roles in the degeneration of human IVDs. During the pathogenesis of IDD, degeneration of the extracellular matrix (ECM) is initiated by proteolytic enzymes, including MMP-1, -3, -7, -9, and -13, as well as ADAMTS-4 and ADAMTS-5^14^,^15^. At a steady state condition, the activity of MMPs is very low in IVD tissue; however, this activity can be up-regulated by inflammatory cytokines^16^. IL-1β, TNF-α, and other pro-inflammatory cytokines are increased in IDD, which induces MMP production and decreases the synthesis of ECM components^17^,^18^,^19^,^20^.

Protease nexin-1(PN-1) is a serine protease inhibitor with a unique structure shared by most serpins, belonging to the serpin superfamily. The reactive center loop of PN-1 is located near the carboxy-terminal end of the serpin domain, which is necessary for its inhibitory activity^21^,^22^. PN-1 can inactivate several proteases, including plasmin, plasminogen, and urokinase, preventing cartilage degradation^23^,^24^,^25^. As is known, the plasmin/plasminogen enzymatic cascades play an important role in cartilage catabolism, which is mediated by activated matrix metalloproteinases (MMP). IVD tissues share pathophysiological characteristics with osteoarthritis (OA)^26^. Various types of proteases are directly involved in ECM degradation; however, MMPs are considered the major enzymes^27^. Given the role of serine proteases in OA pathology, the endogenous serine protease inhibitor PN-1 could share a similar role in ECM degeneration involving the activated plasminogen/plasmin and MMP systems in IVD tissue.

Here, we hypothesize that the expression of PN-1 decreases during IDD, which is related to altered disc-cell function and subsequent characteristic features of IDD. Thus, this study aimed to investigate the expression of PN-1 during IDD, and to determine the induction of its regulation by pro-inflammatory cytokine TNF-α and IL-1β. Then, we examined the effects of PN-1 on the expression and activity of MMPs and ADAMTS in NP cells. Finally, the associated signaling pathway was investigated, focusing on the activation of ERK1/2/NF-κB.

## Materials and Methods

This study complies with accepted ethical standards for human and animal research. The research has been approved by the Ethics Committee of Tongji medical college, and written informed consent was obtained from all participants.

### Materials

Recombinant human PN-1 was purchased from R&D Systems (2980-PI-010). Recombinant human IL-1β (AF-200-01B-10), TNF-α (AF-300-01A-10), and TGFβ1 (AF-100-21C-10) were obtained from Peprotech. Polyclonal antibodies to PN-1 (ab154591), MMP13 (ab39012), ADAMTS5 (ab135656), and Aggrecan (ab3778) were purchased from Abcam. Mouse anti-human fibronectin N-terminal monoclonal antibody (mABl936) was obtained from Chemicon (San Francisco, CA, USA). Polyclonal antibodies against COL2 (sc-7764) and P-P65 (sc-33020) were obtained from Santa Cruz. Polyclonal antibodies against P38 (BS1681), P-P38 (BS6381), P-ERK (BS5016), and MMP9(BS6893) were obtained from Bioworld. P65 (10745-1-AP), and ERK (16443-1-AP) were purchased from ProteinTech Group. The anti-MMP3 (14351) antibody was purchased from CST and the anti-ADAMTS4 (EAP1002) antibody was obtained from Elabscience. All fluorescence-conjugated secondary antibodies, anti-IgG horseradish peroxidase (HRP)-conjugated antibodies, and the LaminB antibody were obtained from BosterBio. DAPI was purchased from Beyotime Biotechnology.

### Patient tissue samples

Degenerated IVD tissues were obtained from 32 symptomatic patients undergoing disc excision and spinal fusion surgery. Non-degenerated disc samples were obtained from the intervertebral discs of eight scoliosis patients undergoing deformity correction surgery. The samples were classified by the degree of IVD degeneration according to the magnetic resonance imaging (MRI) classification of disc degeneration^28^. Degeneration grades were assigned as follows: grade 1–2, non-degenerated (normal disc height); grade 3, mild degeneration (normal to slightly decrease in disc height); grade 4, moderate degeneration (normal of moderately decrease in disc height); and grade 5, severe degeneration (collapsed disc space). Additionally, IVD cryosections from each group were stained with Alcian blue.

### Isolation and Culture of NP Cells

Normal human NP cells were isolated from the discs in younger scoliosis patients undergoing deformity correction surgery (Union Hospital, Tongji Medical College). Briefly, NP tissues were minced into small fragments and enzymatically digested in 0.2% type II collagenase and 0.25% trypsin for 3 hours. After being filtered and washed in PBS, the cells were seeded and cultured in growth medium (DMEM/F-12 supplied with 20% fetal bovine serum, 50 U/mL penicillin, and 50 μg/mL streptomycin) in a 5% CO_2_ incubator. The cells were passaged two to three times for use in the following experiments.

### Stimulation of NP cells

NPs were seeded and cultured in 12-well dishes and allowed to reach 100% confluence. After being serum-starved for 2 hours, the cells were incubated in growth medium alone or in stimulatory medium containing IL-1β (10 ng/mL), TNF-α (50 ng/mL), or TGF-β1 (1 ng/mL) for 7, 14, and 21 days, and the medium was changed every 3 days. In control cultures, the medium was replaced at the indicated times. Each treatment was performed in three different wells. In another experiment, to test the signaling pathways involved in NP cells, PN-1 and/or IL-1β at different concentrations were used to treat cells for 24 h, and inhibitors of NF-kB (Caffeic Acid Phenethyl Ester) and ERK (GDC-0994) were used.

### ELISA

PN-1 is a secreted protein that acts in the ECM, and therefore, the protein analysis was performed on cell-conditioned media. The level of PN-1 was detected by ELISA from the stored supernatant of each group at different time points after induction, according to the manufacture’s procedures (USCN, SED381H).

### Immunohistochemistry

Immunohistochemistry (IHC) for PN-1 expression was performed on degenerated IVD tissue. To detect protein expression, antigen retrieval was performed on the IVD cryosections by incubation in 0.8% hyaluronidase at 37 °C for 60 min. The sections were washed gently with PBS for 5 min, and then blocked in 0.5% goat serum for 40 min at room temperature. Subsequently, the samples were incubated with polyclonal anti-PN-1 (1:100), or control rabbit IgG (2 μg/mL) at 4 °C overnight, followed by washing with PBS. Then secondary antibody conjugated to horseradish peroxidase (diluted 1:3000) was applied for 20~30 min at room temperature. After washing, the sections were incubated with diaminobenzidine (DAB; Solarbio, DA1010) reaction solution until color was detected, followed by counterstaining with hematoxylin, and images were captured using a microscope (Olympus).

### Gene expression analysis

Total RNA was extracted from cells using Trizol reagent (Invitrogen, USA), and reverse transcription was performed to obtain cDNA, according to the manufacturer’s instructions. qPCR was performed using SYBR green (Fermentas, #K0242) and Ex Taq^TM^ (TAKARA, DRR100A) according to manufacturer’s instructions; β-actin was used as an endogenous control. Each 20 μL reaction mixture comprised 4 μL cDNA, 0.4 μL forward primer, 0.4 μL reverse primer, 10 μL SYBR green/flourescein qPCR Master Mix(2X), and 5.2 μL H_2_O. All reactions were performed in triplicate at the following cycling conditions: 95 °C for 10 min, 40 cycles of 95 °C for 30 s, and 60 °C for 30 s. Target mRNA was quantified by real-time RT-PCR and normalized relative to β-actin mRNA according to a standardized protocol. Relative mRNA expression levels were determined by the comparative ΔΔCT method. The oligonucleotide primers for quantification of PN-1, FN, and β-actin mRNA were as follows: Homo PN1, 121bp, F: 5′-CAACTTCATTGAACTGCCCTACC-3′ R: 5′-GCTGTCTATGGTCTTGGTGCTGA-3′; Homo FN1, 209bp F: 5′-AACCTACGGATGACTCGTGCTTT-3′, R: 5′-TTCTCCCTGACGGTCCCACTTCT-3′; Homo β-actin, 285bp F: 5′-AGCGAGCATCCCCCAAAGTT-3′, R: 5′-GGGCACGAAGGCTCATCATT-3′.

Each sample was analyzed in triplicate for both, target and control genes.

### Western Blot Analysis

The NP cells from each culture dish were collected and lysed in RIPA buffer. The lysates were centrifuged, and the protein concentrations were determined by the BCA protein assay. In each group, equivalent amounts of protein (50 μg) were used for electrophoresis. After electrophoresis, the proteins were electrotransferred to 0.45 μm-pore-diameter polyvinylidene difluoride (PVDF) membranes (Invitrogen). After being blocked in Tris-buffered saline containing Tween-20 with 5% milk powder, the membranes were immunolabeled with specific antibodies for PN-1 (1:1500), FN N-terminal (1:5000), P38 (1:800), P-P38 (1:1000), ERK (1:5000), P-ERK (1:800), P65(1:2000), P-P65 (1:800), MMP3(1:1000), MMP9(1:1000), MMP13 (1:4000), ADAMTS4 (1:1000), ADAMTS5 (1:80), Aggrecan (1:100), or COL2 (1:8000) overnight at 4 °C with gentle shaking. After washing, the membranes were incubated for 2 h at 4 °C with a 1:2000 dilution of HRP-conjugated secondary antibody in antibody diluents (Boster). Finally, the ECL Plus western blotting system was used, and immunoreactive bands were quantified using ImageQuant LAS 400 software (GE Healthcare Life Sciences) and calculated by normalization to the reference bands of β-actin or Lamin B.

### Gelatin zymography

Gelatin zymography is a sensitive and quantifiable method for analyzing proteolytic activity of enzymes, including MMP-2 (gelatinase A) and MMP-9 (gelatinase B). In brief, to detect the gelatinolytic activity of MMPs, cell extracts from different groups were incubated at 37 °C for 20 min in SDS sample buffer, and then electrophoresed on 8% polyacrylamide gels at 4 °C. Pre-stained SDS-PAGE markers and MMP-9 and MMP-2 standards were used to estimate the molecular weights of the bands. After electrophoresis, the gels were eluted twice with 2.5% Triton X-100, 50 mmol/L Tris-HCl, and 5 mmol/L CaCl2, pH 7.6 for 40 min. The gels were rinsed with wash buffer without Triton X-100. Subsequently, the gels were incubated with 50 mmol/L Tris-HCl, 5 mmol/L CaCl2, and 0.02% Brij-35, pH 7.6 at 37 °C for 42 h, which allows substrate degradation. The gels were fixed in 30% methanol and 10% acetic acid for 30 min, and stained with 0.5% Coomassie Brillant Blue for 3 h. Proteolytic bands were visualized using destaining solution A, B, and C (30, 20, and 10% methanol, and 10, 10, and 5% acetic acid, respectively). Finally, the gels were scanned, and MMP-9 and MMP-2 proteolytic activities were semiquantified based on computer-assisted image analysis. Results were expressed as the relative percentage of gelatinolytic activity (density of the active band in each group).

### Statistical evaluation

All statistical analyses and plots were performed using the Prism GraphPad 5.0 software. Values are presented as mean ± SEM. All measurements were performed in triplicate. Changes in gene expression between the various treatment groups were evaluated by one-way ANOVA or Student’s *t*-test. A p-value < 0.05 was considered statistically significant.

## Results

### PN-1 gene expression in human NP tissue

**Figure 1:**
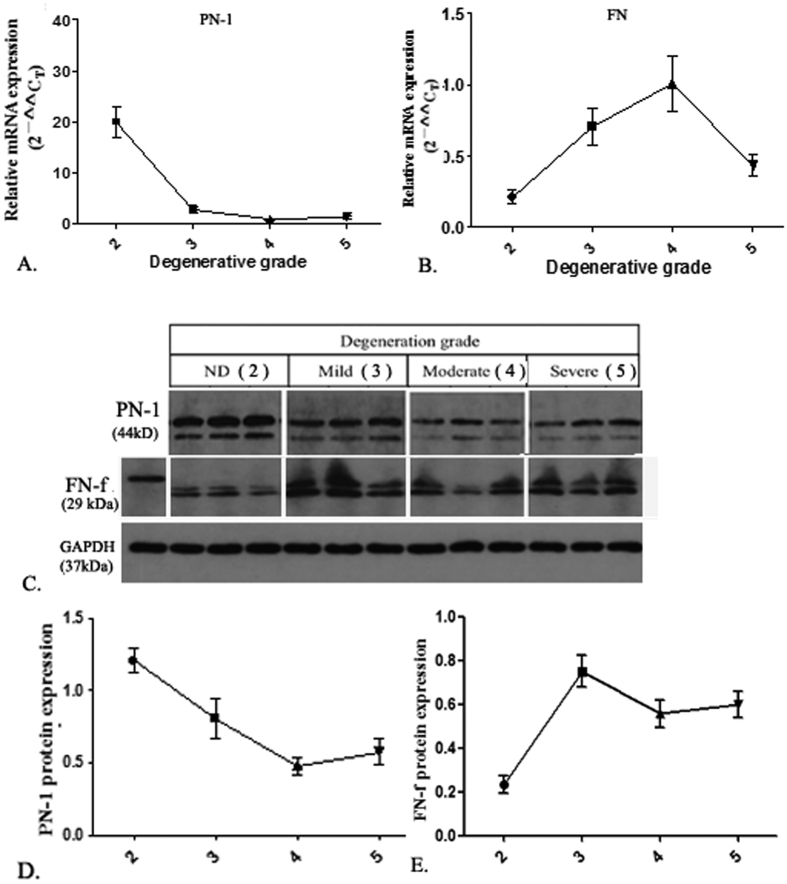
Expression of PN-1 and FN-f mRNA and protein was detected in human IVD tissue. (**A**) PN-1 mRNA levels in human IVD tissue samples (n = 24, 6 samples for each grade of IVD) with varying degrees of IVD degeneration were determined by qPCR. (**B**) FN mRNA expression in different groups as shown by qPCR. (**C**) Representative western blot of PN-1 and FN-fs in human IVD tissue (n = 12, 3 for each grade of IVD) were determined by western blotting methods. (**D,E**) Densitometry analysis of at least three western blot experiments shown in (**C**), PN-1/GAPDH ratio (**D**), and FN-f-1/GAPDH ratio. Data are shown as means ± SD. *p < 0.05, **p < 0.01, compared with Grade II.

PN-1 mRNA expression levels in human IVD tissue with differing grades of disc degeneration were normalized to β-actin and presented as 2^−ΔΔCt^. As seen in Fig. 1A, there was significant expression of PN-1 in non-degenerated IVD tissue (Grade 2), but expression was markedly weakened in degenerated IVD tissue. FN, a protein that induces intervertebral disc degeneration, was increased in the IVD samples with increased degrees of disc degeneration (Fig. 1B). Western blot analysis was performed to detect the protein expression of PN-1, FN, and FN-fs in IVD with varying degrees of disc degeneration (Fig. 1C). As the photomicrographs show, the expression of PN-1 was similar to that seen by PCR (Fig. 1D), indicating that gene expression of PN-1 decreased during the process of disc degeneration. FN-fs play important roles in the development of human disc degeneration. In the study, FN and FN-f were analyzed by western blot, which showed that the highest amount of FN-fs emerged in the moderately degenerative discs (grades III) and retained a high level during IDD (Fig. 1E).

### Histological examination for PN-1 expression in human NP tissue

**Figure 2:**
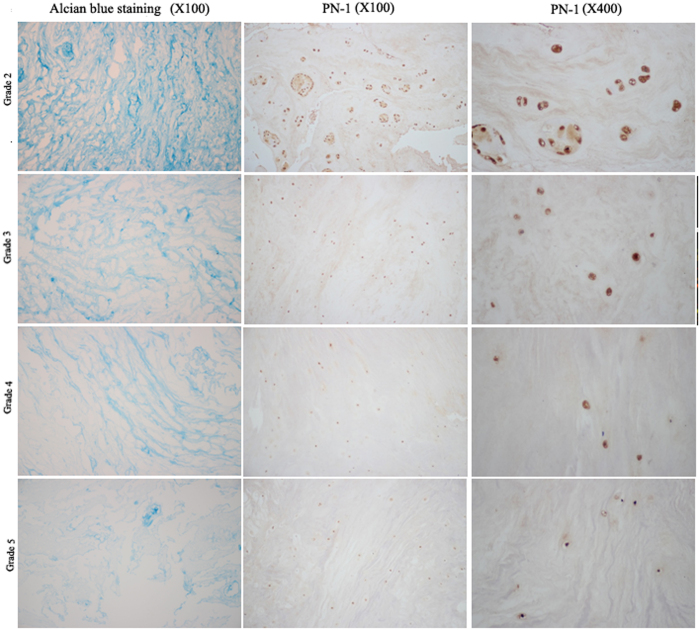
PN-1 protein expression in human IVD tissue. Representative images of PN-1 protein from paraffin-embedded IVD tissue sections (n = 12, 3 for each grade of IVD) that was detected by immunohistochemistry staining. Grade 2 represents a non-degenerated IVD, whereas grades 3, 4, and 5 signify mild, moderate, and severe degeneration, respectively. (Alcian blue staining 100×; IHC 100×, 400×).

The degree of degeneration of the IVD tissue was confirmed by Alcian blue staining. IHC was used to confirm the expression of PN-1 in human IVD tissue. As shown in Fig. 2, the extent of PN-1 expression by NP cells was clearly decreased in the degenerated IVD sample. Thus, the expression of PN-1 is inversely correlated with the grade of disc degeneration, in that a higher degenerative degree correlated to a lower expression level of PN-1 (Fig. 2).

### PN-1 Expression in NP cells in response to IL-1β stimulation

**Figure 3:**
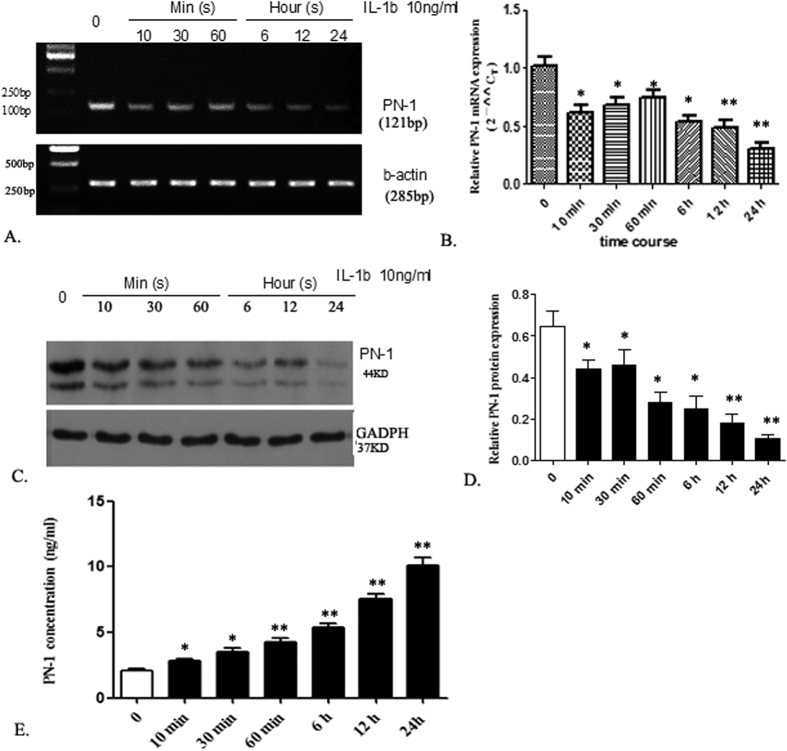
IL-1β decreases PN-1 expression in NP Cells. (**A**) RT-PCR analysis of NP cells treated with IL-1β for 10, 30, and 60 min, for 6, 12, and 24 h. (**B**) PN-1 mRNA levels were determined by qRT-PCR and presented as 2^−△△Ct^. *p < 0.05, **p < 0.01, determined by one-way ANOVA. (**C**) Western blot analysis of cell lysates of NP cells treated with IL-1β, showing PN-1 protein expression. (**D**) Densitometry analysis of western blot experiments as shown in (**C**), showing a decrease in PN-1 protein level. (**E**) ELISA of conditioned cell media from NP cells treated with IL-1β, showing an increase in PN-1 protein expression. Data are presented as means ± SD, from at least three independent experiments. *p < 0.05, **p < 0.01, compared with the control group.

To determine the expression of PN-1 mRNA at the cellular level under degeneration, NP cells were cultured with IL-1β, and mRNA was detected using RT-PCR at different time points (10, 30, and 60 min, and 6, 12, and 24 h). As Fig. 3 shows, administration of IL-1β resulted in a significant decrease in PN-1 mRNA expression at 24 h (Fig. 3A,B). Cell lysates from different time points were collected and analyzed by western blot, which also confirmed a significant decrease of PN-1 expression at 24 h (Fig. 3C,D). The analysis of PN-1 expression levels on cell-conditioned media was performed by an ELISA, which indicated that PN-1 concentration increased significantly in NP cells in a time-dependent manner after IL-1β induction (Fig. 3E). Therefore, IL-1β induction could decrease the gene expression of PN-1 in NP cells, and the decrease in cytosolic PN-1 protein expression is due to an increase in protein secretion in the culture media.

### Pro-inflammatory cytokines IL-1β and TNF-α decrease PN-1 expression in NP cells

**Figure 4:**
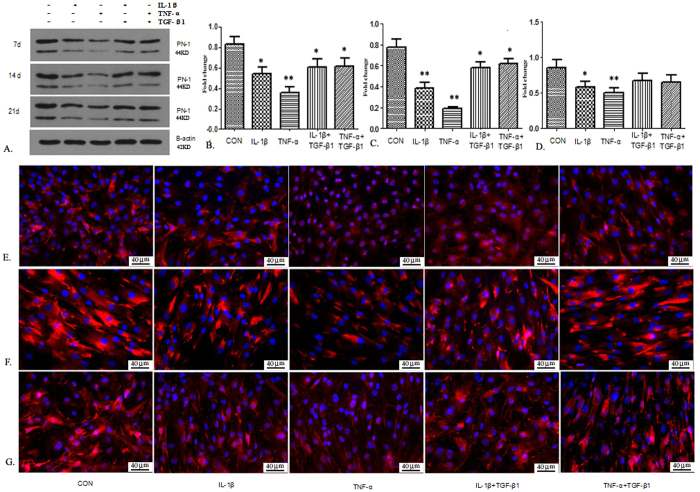
Analysis of immunopositivity for PN-1 expression in NP cells in the presence and absence of pro-inflammatory cytokines. (**A**) The protein expression of PN-1 in NP cells was determined after stimulation with IL-1β, TNF-α, IL-1β + TGF-β1, and TNF-α + TGF-β1. (**B**) Quantitative analysis of immunopositivity for NP-1 at day 7. (**C**) Quantitative analysis of immunopositivity for NP-1 at day 14. (**D**) Quantitative analysis of immunopositivity for NP-1 at day 21. (**E**–**G**) Representative photomicrographs of immunohistochemical staining for PN-1 expression in NP cells at different time points: 7 days (**E**), 14 days (**F**), and 21 days (**G**). Four randomly selected fields (up–down–left–right) were analyzed for each sample. *p < 0.05, **p < 0.01, compared with the control group. (Scale bar magnification: 400×).

To detect the expression of PN-1 regulated by IL-1β and TNF-α, human NP cells were cultured with IL-1β (10 ng/mL), TNF-α (50 ng/mL), IL-1β+TGF-β1 (10 ng/mL), and TNF-α+TGF-β1, and detected by western blotting and immunofluorescent staining. The protein expression levels in different groups were examined at different time points (Fig. 4A). Western blot analysis showed a significant decrease in the PN-1 protein level in NP cells after administration of IL-1β and TNF-α on days 7 (Fig. 4B) and 14 (Fig. 4C). There was still a trend of decreased PN-1 expression at day 21 (Fig. 1D), but it was not as evident as on days 7 and 14. The results also indicated that TGF-β1 treatment could alleviate the suppressive effects of pro-inflammatory cytokines. Additionally, immunofluorescent staining for PN-1 expression was performed in cells cultured with IL-1β and TNF-α at the corresponding time points, which confirmed the decreased expression of PN-1 in NP cells in comparison with the control group at day 7 (Fig. 4E), 14 (Fig. 4F), and 21 (Fig. 4G).

### PN-1 inhibits IL-1β-induced MMP production in NP cells

**Figure 5:**
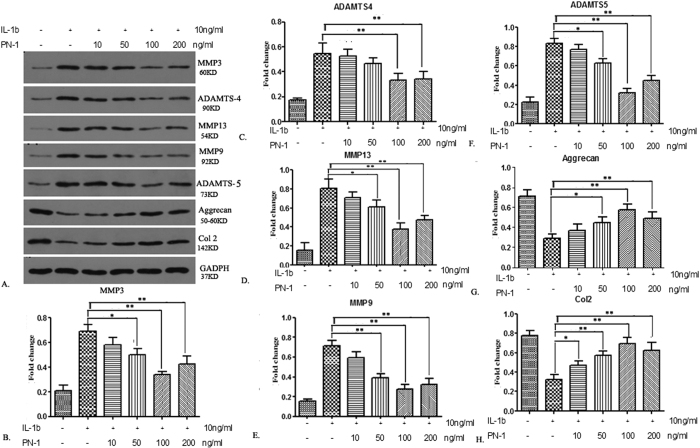
Comparison of immunoblotting for quantitative analysis of relative protein change in NP cells. Cells were treated with IL-1β (10 ng/mL) alone or combined with PN-1 at different concentrations (0, 10, 50, 100, or 200 ng/mL). (**A**) Equal amounts of protein extract were resolved by SDS-PAGE, and MMP-3, MMP-9, MMP-13, ADAMTS-4, ADAMTS-5, aggrecan, and COL2 proteins were detected by western blot analysis. (**B**–**H**) Densitometric analysis shows the suppression of MMP3 (**B**), ADAMTS-4 (**C**), MMP-13 (**D**), MMP-9 (**E**), and ADAMTS-5 (**F**) Protein levels in NP cells treated by IL-1β and PN-1. (**G,H**) Densitometric analysis shows the promotion effect of Aggrecan (**G**) and COL2 (**H**) Protein levels in NP cells treated by IL-1β and PN-1. Data are representative of three independent experiments, and p-values are shown: *p < 0.05; **p < 0.01 compared to IL-1β stimulation alone.

To investigate the effects of PN-1 on the protein expression of matrix degrading enzymes in IVD, NP cells were cultured in the presence of IL-1β with or without PN-1, and proteins were detected by western blotting. Changes in MMP-3, ADAMTS-4, MMP-13, MMP-9, ADAMTS-5, Agg, and COL2 levels were observed (Fig. 5A). Specifically, IL-1β treatment resulted in a significant up-regulation of MMP protein expression after 24 h stimulation in human NP cells. Interestingly, recombinant PN-1 was able to reverse the production of MMPs induced by IL-1β in human NP cells. Compared to IL-1β stimulation alone, PN-1 administration could reverse the expression of MMP-3 (Fig. 5B), ADAMTS-4 (Fig. 5C), MMP-13 (Fig. 5D), MMP-9 (Fig. 5E), and ADAMTS-5 (Fig. 5F) in a dose-dependent manner. The optimal concentration of PN-1 was 100 ng/mL. We detected the expressions of Agg and COL2. The results showed that IL-1β treatment down-regulated expressions of Agg and COL2, while PN-1 administration could attenuate the effects, reaching the highest levels at the optimal dose of 100 ng/mL.

### PN-1 inhibits IL-1β-induced gelatinolytic activities of MMPs

**Figure 6:**
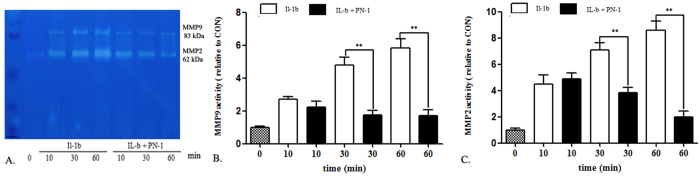
Gelatinolytic activity of matrix metalloproteinase-2 and -9 in NP cells. (**A**) Molecular markers indicate active MMP-9 and -2 as 92 and 62 kDa proteins, respectively. (**B**) Zymographic band densities for MMP-9 were quantified. (**C**) Zymographic band densities for MMP-2 were quantified. Data are representative of three independent experiments, and p-values are shown: *p < 0.05; **p < 0.01, compared to IL-1β stimulation alone.

MMPs are enzymes that degrade components of the ECM, and among them, MMP-2 and -9 are the most effective gelatinolytic MMPs. Cell gelatinolytic activity in conditioned cell media has been performed successfully. The zymographic pattern (Fig. 6A) revealed gelatinolytic activity in different groups, expected for MMP-2 and MMP-9. The active band for MMP-9 (83 kDa) was reduced 0.36 and 0.29 fold after PN-1 administration, compared to after administration of IL-1β alone, at 30 and 60 min (Fig. 6B). Similarly, MMP-2 (62 kDa) was found to be reduced 0.54 and 0.22 fold (Fig. 6C). These results indicated that IL-1β causes a significant up-regulation of MMP-9/2 expression and gelatinolytic activity, while exogeneous PN-1 could invert the effects in human NP cells.

### PN-1 inhibits IL-1β-induced MMP production through ERK1/2/NF-κB activation in NP cells

**Figure 7:**
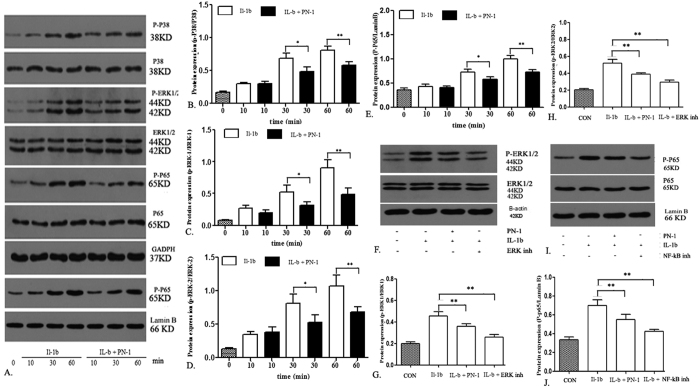
The suppressive effect of PN-1 on the IL-1β-induced NF-B pathway. (**A**) NP cells were stimulated with IL-1β alone or combined with PN-1. Cells from different groups were collected and whole-cell protein was extracted at 10, 30, and 60 min time points. The phosphorylated and unphosphorylated levels of P38, ERK1/2, and P65 were detected by western blot analysis. (**B**–**E**) Densitometric analysis shows the suppression of P-P38 (**B**), P-ERK-1 (**C**), P-ERK-2 (**D**), and P-P65 (**E**) protein levels in NP cells treated with IL-1β and PN-1. F. NP cells were stimulated with IL-1β alone or in combination with PN-1 or ERK inhibitor. The phosphorylated and unphosphorylated levels of ERK1/2 were detected by western blot analysis. (**G**–**H**) Densitometric analysis shows the suppression of P-ERK-1 (**G**) and P-ERK-2 (**H**) protein levels in NP cells treated with IL-1β alone or combined with PN-1 or ERK inhibitor. (**I**) NP cells were stimulated with IL-1β alone or combined with PN-1 or NF-kB inhibitor. The levels of phosphorylated and unphosphorylated P65 were detected by western blot analysis. Nucleoprotein extracts were prepared and analyzed for the expression of P-P65. (**J**) Densitometric analysis shows the suppression of P-P65 protein levels in NP cells treated with IL-1β alone or combined with PN-1 or NF-kB inhibitors. Data were obtained from three independent experiments. GADPH/Lamin B were used as the loading control. *p < 0.05; **p < 0.01, compared to IL-1β stimulation alone.

To investigate the effects of PN-1 on the signaling pathway of IL-1β-induced IVD degeneration, we treated human NP cells with IL-1β, with or without specific inhibitors of NF-kB (Caffeic Acid Phenethyl Ester) and ERK (GDC-0994). Levels of phosphorylated and unphosphorylated P38, ERK1/2, and P65 proteins were determined by western blotting (Fig. 7). The levels of unphosphorylated P38 were similar in both groups at different time points. IL-1β stimulation induced increased levels of phosphorylated p38, which was significantly inhibited in NP cells treated by recombinant PN-1 addition (Fig. 7B). ERK1 and ERK2 contribute to cytokine dependent induction of intervertebral disc degeneration, and hence, activate the inflammatory-related signaling molecule NF-κB. To obtain further insights into the underlying mechanism, NF-κB signaling was also investigated. We observed that IL-1β induced increased levels of phosphorylated ERK1/2, which was significantly blocked by the addition of recombinant PN-1 (Fig. 7C,D). Hence, we sought to analyze whether PN-1 was able to modulate the phosphorylated levels of downstream effector P65, besides NF-κB. As shown in Fig. 7E, and as expected, IL-1β was able to increase the phosphorylated levels of P65, and the addition of recombinant PN-1 reduced the phosphorylated levels of P65. Finally, an ERK inhibitor was used, which showed that the phosphorylated levels of ERK1/2 were partially blocked by recombinant PN-1 in NP cells (Fig. 7G,H); an NF-kB inhibitor was also used, and as expected, the results showed that levels of P-P65 were significantly blocked by PN-1 addition. Therefore, the ERK1/2/NF-κB signaling pathways were involved in the regulation of PN-1 in the pathogenesis of IVD degeneration.

### PN-1 inhibits IL-1β-induced MMP production through ERK1/2/NF-κB signal pathways

**Figure 8:**
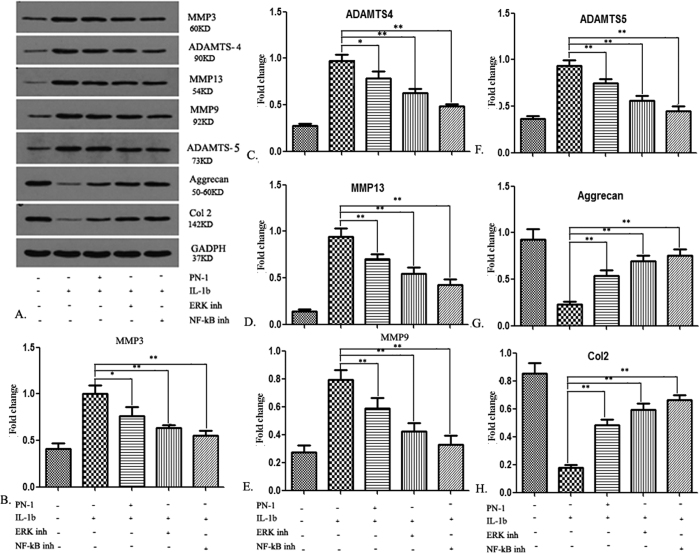
Down-regulation of IL-1β-induced MMP expression in NP cells by PN-1. Cells were treated with IL-1β (10 ng/mL) alone or combined with PN-1, ERK inhibitor, or NF-kB inhibitor. (**A**) Equal amounts of protein extract were resolved by SDS-PAGE, and MMP-3, MMP-9, MMP-13, ADAMTS-4, ADAMTS-5, aggrecan, and COL2 proteins were detected by western blot analysis. (**B**–**F**) Densitometric analysis shows the suppression of MMP3 (**B**), ADAMTS-4 (**C**), MMP-13 (**D**), MMP-9 (**E**), and ADAMTS-5 **(F**) protein levels in NP cells treated with IL-1β, IL-1β + PN-1, IL-1β + ERK inhibitor, and IL-1β + NF-kB inhibitor. Densitometric analysis shows the promotive effect on Aggrecan (**G**) and COL2 (**H**) protein levels in NP cells treated with IL-1β, IL-1β + PN-1, IL-1β + ERK inhibitor, and IL-1β + NF-kB inhibitor. Data are representative of three independent experiments, and p-values are shown: *p < 0.05; **p < 0.01 compared to IL-1β stimulation alone.

To investigate the effect of the PN-1 signaling pathway on the expression of matrix degrading enzymes in IVD, NP cells were cultured in the presence of IL-1β with or without specific inhibitors of NF-kB (Caffeic Acid Phenethyl Ester) and ERK (GDC-0994), and MMP proteins were detected by western blotting (Fig. 8A). Specifically, IL-1β administration could up-regulate the production of MMPs in NP cells after stimulation with PN-1, ERK inhibitor or NF-kB inhibitor. MMP-3 (Fig. 8B), ADAMTS-4 (Fig. 8C), MMP-13 (Fig. 8D), MMP-9 (Fig. 8E), and ADAMTS-5 (Fig. 8F) levels were significantly inhibited. Meanwhile, expressions of Agg (Fig. 8G) and COL2 (Fig. 8H) were up-regulated for the corresponding groups. Therefore, the results showed that IL-1β could up-regulate the expression of MMPs through both ERK1/2/NF-κB signaling pathways in NP cells, which could be suppressed by exogenous PN-1.

## Discussion

Our findings from clinical samples provide evidence for our hypothesis that PN-1 is expressed in human IVD tissue and is highly regulated during the process of IDD *in vivo* and *in vitro*. These results also indicate that PN-1 could inhibit the activation of MMPs and ADAMTS through the ERK1/2/NF-κB signaling pathway. In all, these findings provide insights into the molecular mechanisms of IDD. Pro-inflammatory cytokines such as IL-1β and TNF-α, influence the degeneration of intervertebral discs, through protease pathways.

PN-1, a 45- to 50-kDa glycoprotein, is encoded by the *SERPINE2* gene on human chromosome 2q99-q35^29^. PN-1 is a member of the serine protease inhibitor (serpin) family, involved in tissue remodeling, cellular invasion, matrix degradation, and tumor growth^23^. PN-1 is barely detectable in plasma, but is found in many organs, such as the brain, heart, kidneys, lungs, spleen, muscle, and cartilage^30^,^31^, and is produced by most cell types, such as glial cells^32^, smooth muscle cells (SMCs)^33^, endothelial cells^34^, and fibroblasts^35^. PN-1 primarily localizes at the cell surface by binding to glycosaminoglycans (GAGs)^36^, which trap and potentiate PN-1 activity within the pericellular space, so that proteolysis does not lead to widespread protein destruction^21^,^37^,^38^. Therefore, the activity of proteases on cells is regulated by PN-1 bound to cell-surface GAGs. Furthermore, PN-1 plays an important role in the process of proteolysis in an irreversible and highly complex manner through the inhibition of proteases^39^.

Osteoarthritis (OA), a joint disease caused by cartilage loss, is strongly associated with a net loss of aggrecan and collagen breakdown caused by an imbalance in ECM homeostasis^40^. The function of articular cartilage depends on the molecular composition of the ECM, which mainly consists of collagen II/IX/XI fibrils and proteoglycan- glycosaminoglycan networks of aggrecan and hyaluronan. ECM components are essential in regulating chondrocyte metabolism and function, and play a crucial role in the ability of the tissue to withstand compressive forces and respond to mechanical loading^40^. In the cartilage of OA patients, there is a significant increase in the level of serine protease Htra1 (high temperature factor A-1), which mediates proteolysis of aggrecan and contributes to OA pathology^41^,^42^. Initially, PN-1 was identified as a serine protease inhibitor in cartilage anlagen during embryogenesis, and interacted with both ECM and intracellular cartilage components^30^,^43^,^44^, which is consistent with its role in the binding and inactivation of extracellular proteases followed by internalization^45^. The activity of PN-1 is regulated by several matrix components, such as FN^46^, collagen II, and collagen I^47^. Therefore, serine proteases play an important role in cartilage pathology, and endogenous serine protease inhibitors such as PN-1 could potentially be promising new therapeutic targets in OA^43^,^48^,^49^.

IVD tissues share pathophysiological characteristics with OA^26^; however, little is known about the involvement of serine proteases in the pathogenesis of disc degeneration. Type I and II collagen fibrils make up almost 20% and 70% of the collagen network in the dry weight of the nucleus pulposus and annulus fibrosus, respectively^50^. Previous studies have confirmed that IDD is associated with fibrosis of the NP, in which the collagen meshwork is gradually replaced by fibrils of abnormal size and rigidity^6^, resulting in altered gene expression for matrix molecules, degradation enzymes, and catabolic cytokines^18^,^51^. In addition, alterations in PG lead to a decrease in hydration, loss of structural integrity, and an inability to withstand loads^52^,^53^. The molecular mechanisms of collagen fibrilogenesis are complicated, but highly related to mechanical stress, collagen genetic types, and aging^54^. Data are available for structural proteins of IVD, such as aggrecan^55^ and collagen^56^; however, little is known about the longevity of serine proteases from this tissue. The imbalance between serine proteases and serpins may have an effect on the degeneration of IVD; thus, expression of PN-1 is important to gain further insight into the mechanisms of IDD.

The present study was designed to investigate the changes of PN-1 expression in degenerated IVD of different grades using RT-PCR and IHC. Investigations in human IVD tissue have demonstrated a decreased expression of PN-1 following IDD. Moreover, we found that PN-1 mRNA and protein levels were coordinately regulated during the progression of IDD in a stage-specific manner. Inflammatory cytokines, such as IL-1β and TNF-α, have been known to induce degeneration of IVD. Based on *in vitro* experiments, changes in MMP protein and mRNA expression levels have been detected by western blotting and RT-PCR, and activity of MMPs have been examined by gelatin zymography. IL-1β, TNF-α, and other pro-inflammatory cytokines have also been shown to induce MMP production in NP cells. As our results indicated, when stimulated with IL-1β, the secretion of MMPs and ADAMTS by human NP cells increased, whereas PN-1 administration could antagonize these proteases and potentially preserve IVD tissue from degeneration.

Fibronectin (FN) and its fragments (FN-fs) have been found to accumulate during disc degeneration and acceleration of IVD degeneration in rabbits^57^. Within human degenerated-disc tissue samples, a marked increase in FN and FN-fs has been observed; thus, they are considered an integral part of the underlying pathology of IDD^58^,^59^,^60^. PN-1 activity is at least partly regulated by the extracellular matrix component fibronectin^46^,^61^, and fibronectin and its fragments have been observed to be potent inducers of MMP expression during IVD degeneration^62^. In addition, serine proteases have been confirmed to regulate MMP expression on the generated fibronectin fragments^59^. Therefore, an endogenous serine protease inhibitor, such as PN-1, has potentially roles in modulating MMP expression in IVD cells. Nuclear factor kappa B (NF- κB) is a family of transcription factors, which becomes activated in response to inflammation, damage, and stress^63^,^64^. MMPs have been identified as NF- κB target genes in IVD cells responsible for ECM degradation, including MMP-1, MMP-2, MMP-3, MMP-9, and MMP-13^15^,^59^,^65^. NF-κB play a central role in MMP and ADAMTS expression in NP cells stimulated by IL-1β^66^. Activation of the NF-κB signaling pathway results in increased matrix-degrading enzyme activity in the NP, which is an important catabolic pathway involved in IDD^67^. Therefore, NF- κ B pathway has been fully investigated in human NP cells stimulated with PN-1.

Disc degeneration is characterized by the loss of Agg and COL2^68^. Agg is a type of proteoglycan responsible for the normal structure of discs, while COL2 is an important component of the ECM, helping the IVD bear pressure. In this study, IVD degeneration induced by IL-1β lead to loss of Agg and COL2 in NP cells, which was associated with upregulation of MMPs and ADAMTS. PN-1 intervention reversed the downregulation of Agg and COL2 and the upregulation of MMPs, implying that PN-1 could partially antagonize NF-κB pathway-specific transcription factors and contribute to maintaining matrix integrity of the IVD. Moreover, we found that PN-1 could inhibit IL-1β-induced activation of ERK1/2 in NP cells. Therefore, PN-1 intervention can potentially reverse the expression of MMPs and ADAMTS through the ERK1/2/NF-κB signaling pathway in IDD. Agg and COL2 are necessary for the rebuilding of IVD, which also can be upregulated through the pathway by PN-1.

In conclusion, under inflammatory conditions, activated ERK1/2/NF-κB contributes to the pathogenesis of IDD, manifested by the upregulation of MMPs and ADAMTS and degraded disc matrix macromolecules Agg and collagen II. PN-1 is involved in pathogenesis, at least in part by suppressing IL-1β-induced activation of ERK1/2/NF-κB and its downstream targets. Thus, decreased expression of PN-1 may serve as an important endogenous mechanism to increase the deleterious effects of excessive serine protease activity within the IVD.

## Additional Information

**How to cite this article**: Wu, X. *et al*. The Involvement of Protease Nexin-1 (PN1) in the Pathogenesis of Intervertebral Disc (IVD) Degeneration. *Sci. Rep.* **6**, 30563; doi: 10.1038/srep30563 (2016).
